# Complete Genome Sequence of a New Member of the *Marseilleviridae* Recovered from the Brackish Submarine Spring in the Cassis Port-Miou Calanque, France

**DOI:** 10.1128/genomeA.01148-15

**Published:** 2015-11-25

**Authors:** Gabriel Doutre, Bruno Arfib, Pierre Rochette, Jean-Michel Claverie, Patricia Bonin, Chantal Abergel

**Affiliations:** a Structural and Genomic Information Laboratory (IGS), Aix-Marseille Université, CNRS UMR 7256 (IMM FR 3479), Marseille, France; b Aix-Marseille Université, CNRS, IRD, CEREGE UM34, ECCOREV, Aix en Provence, France; c Assistance Publique des Hôpitaux de Marseille (APHM), Marseille, France; d Mediterranean Institute of Oceanology (MIO), Aix-Marseille Université, Université de Toulon, CNRS/INSU, IRD, UM 110, Marseille, France

## Abstract

*Marseilleviridae* is a rapidly expanding family of *Acanthamoeba*-infecting large DNA viruses distributed worldwide. We report here the complete 349-kbp genome sequence of Port-Miou virus, which is surprisingly close to that of Lausannevirus (isolated from the Seine River upstream from Paris, France), despite the strong dissimilarities of their sampling locations.

## GENOME ANNOUNCEMENT

The origin of the brackish water flowing from a submarine karstic spring in Port-Miou (Cassis, France) remains enigmatic, although it has been investigated by underwater exploration and various geochemical methods since the 1950s (1). It has been postulated that seawater is introduced very far into the karst and mixed with the freshwater system at a depth of >223 m below sea level (1, 2). To investigate this hypothesis, we recently initiated a comparison of the microbiomes of various samples from possible marine and inland water sources. In this context, we isolated a new representative of the *Marseilleviridae* family of *Acanthamoeba*-infecting large DNA viruses (3, 4).

Eight liters of water collected in the underwater karst conduit (approximately 500 m inside from its mouth) was filtered through 0.8-µm-pore-size nitrocellulose membranes (Millipore). These membranes were then used to seed *Acanthamoeba* cultures, as previously described (5). Following the visual detection of cell lysis, virus particles were amplified and purified on a sucrose gradient (5).

Seven micrograms of purified viral DNA was used to generate paired-end sequence (2 × 300-bp) reads using the MiSeq platform, according to the manufacturer protocol (V3 kit). Following the removal of adapter and cloning vector sequences and the trimming of low-quality read ends (*Q* < 28) using Trimmomatic (6), 1.61 M high-quality paired-end reads were assembled with IDBA-UD (7), resulting in 9 initial contigs that were readily assembled into a single contig using Phrap (8). The read pairs were mapped onto scaffolds using Bowtie (9), and sequencing coverage levels were estimated for each scaffold using SAMtools (10).

The final 349,275-bp sequence (assembled with a high average coverage of 1,213×) is predicted to encode 410 proteins, all of them with highly similar (>90% identical) *Marseilleviridae* homologs. Moreover, the genome of this new representative, named Port-Miou virus, is amazingly close to that of Lausannevirus (346,754 bp) (11). The two sequences are almost perfectly collinear, with their pairwise alignment exhibiting approximately 1 indel/1,000 bp and 99% identical nucleotides among the aligned positions. The most noticeable difference lies in the 4212 to 7452 interval coding for a helicase (PMV_037) and that is most similar (91% identity among 1,088 residues) to a homolog in insectomime virus (12) (ISTM_389, accession no. AHA46375). The corresponding Lausannevirus segment is inverted and encodes a more divergent homolog. Port-Miou virus also encodes several additional endonuclease-like proteins, probably parts of mobile elements, the homologs of which are absent at the corresponding positions in Lausannevirus. The *Marseilleviridae* are presently divided into 3 subclades (13, 14). Port-Miou virus is now the second member of the B subclade, which previously contained Lausannevirus only. Following Melbournevirus (a member of the A subclade), the amazing similarity of Port-Miou virus with Lausannevirus further illustrates the puzzling genomic stability of spatially distant and ecologically distinct *Marseilleviridae* isolates (5). Lausannevirus was never handled in our laboratory, thus ruling out the possibility that Port-Miou virus originated from a contamination event.

### Nucleotide sequence accession number.

The completely annotated genomic sequence of Port-Miou virus has been deposited in GenBank under the accession no. KT428292.
